# Kunitz-Type Peptides from Sea Anemones Protect Neuronal Cells against Parkinson’s Disease Inductors via Inhibition of ROS Production and ATP-Induced P2X7 Receptor Activation

**DOI:** 10.3390/ijms23095115

**Published:** 2022-05-04

**Authors:** Aleksandra Kvetkina, Evgeny Pislyagin, Ekaterina Menchinskaya, Ekaterina Yurchenko, Rimma Kalina, Sergei Kozlovskiy, Leonid Kaluzhskiy, Alexander Menshov, Natalia Kim, Steve Peigneur, Jan Tytgat, Alexis Ivanov, Naira Ayvazyan, Elena Leychenko, Dmitry Aminin

**Affiliations:** 1 G.B. Elyakov Pacific Institute of Bioorganic Chemistry, Far Eastern Branch, Russian Academy of Sciences, 690022 Vladivostok, Russia; kvetkinaan@gmail.com (A.K.); pislyagin@hotmail.com (E.P.); ekaterinamenchinskaya@gmail.com (E.M.); eyurch@piboc.dvo.ru (E.Y.); kalinarimma@gmail.com (R.K.); sergeimerx@gmail.com (S.K.); almenshov1990@gmail.com (A.M.); natalya_kim@mail.ru (N.K.); leychenko@gmail.com (E.L.); 2 V.N. Orekhovich Institute of Biomedical Chemistry, 10, Pogodinskaya St., 119121 Moscow, Russia; leonid.kaluzhskiy@ibmc.msk.ru (L.K.); professor-ivanov@yandex.ru (A.I.); 3 Toxicology and Pharmacology, Campus Gasthuisberg O&N2, University of Leuven (KU Leuven), Herestraat 49, P.O. Box 922, B-3000 Leuven, Belgium; steve.peigneur@kuleuven.be (S.P.); jan.tytgat@kuleuven.be (J.T.); 4 L.A. Orbeli Institute of Physiology, National Academy of Sciences of Armenia, Yerevan 0028, Armenia; taipan@ysu.am

**Keywords:** Kunitz-type peptides, neuroprotective activity, sea anemones, TRPV1, P2X7R, Parkinson’s disease (PD)

## Abstract

Parkinson’s disease (PD) is a socially significant disease, during the development of which oxidative stress and inflammation play a significant role. Here, we studied the neuroprotective effects of four Kunitz-type peptides from *Heteractis crispa* and *Heteractis magnifica* sea anemones against PD inductors. The peptide HCIQ1c9, which was obtained for the first time, inhibited trypsin less than other peptides due to unfavorable interactions of Arg17 with Lys43 in the enzyme. Its activity was reduced by up to 70% over the temperature range of 60–100 °C, while HCIQ2c1, HCIQ4c7, and HMIQ3c1 retained their conformation and stayed active up to 90–100 °C. All studied peptides inhibited paraquat- and rotenone-induced intracellular ROS formation, in particular NO, and scavenged free radicals outside the cells. The peptides did not modulate the TRPV1 channels but they affected the P2X7R, both of which are considered therapeutic targets in Parkinson’s disease. HMIQ3c1 and HCIQ4c7 almost completely inhibited the ATP-induced uptake of YO-PRO-1 dye in Neuro-2a cells through P2X7 ion channels and significantly reduced the stable calcium response in these cells. The complex formation of the peptides with the P2X7R extracellular domain was determined via SPR analysis. Thus, these peptides may be considered promising compounds to protect neuronal cells against PD inductors, which act as ROS production inhibitors and partially act as ATP-induced P2X7R activation inhibitors.

## 1. Introduction

Parkinson’s disease (PD) is a common neurodegenerative disorder associated with dopaminergic neuron losses in the *substantia nigra*, resulting in such symptoms as resting tremor, muscular rigidity, and hypokinesia [1,2]. Numerous studies have shown that oxidative stress and neuroinflammation are key factors in the development and maintenance of the progressive neurodegeneration process in this disease. The main characteristics of oxidative stress are increased levels of reactive oxygen species (ROS) and a decrease in, or dysfunction of, the antioxidant systems to counter free radicals [3,4,5]. The neurotoxins that produce irreversible PD-related effects via oxidative stress induction include paraquat, rotenone, 6-hydroxydopamine (6-OHDA), 1-methyl-4-phenyl-1,2,3,6-tetrahydropyridine (MPTP), and its active metabolite methyl-4-phenylpyridine (MPP^+^), which are the most widespread compounds [6]. All of these compounds exhibit neurotoxic activity due to the ROS formation inside the cells, including the inhibition of mitochondrial complex I, resulting in depletion of intracellular adenosine-5’-triphosphate (ATP) and cell death [7,8,9,10,11,12]. An exceptional feature is characterized for 6-OHDA, which is able to appear in catecholaminergic neurons and is rapidly and nonenzymatically oxidized by molecular oxygen into hydrogen peroxide and p-quinone [13]. 

Oxidative stress can modulate mechanisms involving activation of transient receptor potential (TRP) channels, in particular transient receptor potential vanilloid 1 (TRPV1) [14], and inflammatory response by the activation of signaling pathways, which induce the secretion of a high level of proinflammatory mediators [15,16] and is also accompanied by neuron damage [17]. Cell death tends to expand the consequences of neuroinflammatory processes via the release of biomolecules, such as ATP, activating purinergic receptors, including P2X7 (P2X7R). P2X7R is widely expressed in the regions of the central nervous system, such as the frontal cortex, hippocampus, amygdala, and striatum, involved in neurodegenerative diseases [18]. This receptor and its ligands play important roles in inflammation, tumorigenesis, development, and metastasis [19,20]. The activation of P2X7R by high concentrations of extracellular ATP results in the rapid influx of Na^+^ and Ca^2+^ and efflux of K^+^ [21]. Long-term activation induces the formation of non-selective pores, allowing the influx of the organic ions and fluorescent dyes up to 900 Da, which ultimately leads to cell death and the release of new portions of ATP in the extracellular milieu [22,23]. Therefore, the application of P2X7R antagonists will probably attenuate the processes of neuroinflammation and neurodegeneration [24,25,26].

Understanding the cellular mechanisms the make neurons vulnerable is one of the primary research directions [27]. At present, low-molecular antioxidants, dopamine precursors, and dopamine agonists are widely used for PD symptom elimination rather than delaying the degeneration of dopaminergic neuron [1]. Some peptides and proteins have been shown to reduce the destruction of dopamine neurons induced by oxidative stress [28,29,30]. In this context, peptides of the Kunitz/BPTI family are of great research interest. This structural group of peptides known as protease inhibitors is well-characterized and widespread among both venomous terrestrial and marine animals [31]. In snakes, spiders, scorpions, frogs, cone snails, and sea anemones, Kunitz peptides are encoded by multigene families and form a great diversity of peptide isoforms in the venomous secretion [32,33,34,35,36,37]. The primary function of most known Kunitz-type peptides is protease inhibition. Their structure is stabilized by three conserved intradomain disulfide bonds (CysI–CysVI, CysII–CysIV, CysIII–CysV) accompanied by the formation of two loops responsible for protease inhibition. Amino acid (a.a.) residues of the main protease-binding loop, in particular residues of reactive sites (P1-P1’ positions), form the most contacts with the protease active site and contribute to the association energy of the protein–enzyme complex [38]. The presence of mutations in Kunitz-type peptides does not affect the spatial molecule structure but leads to the appearance of diverse biological activities toward various targets [39,40,41,42]. Recently, we demonstrated that Kunitz-type peptides of the sea anemone *Heteractis crispa* inhibit neuroblastoma cell death induced by 6-OHDA via ROS production reductions or antiradical activity [43,44]. Furthermore, some of them demonstrated anti-inflammatory, antihistamine, and analgesic activities [45,46,47,48,49,50], which might also facilitate decreases in both inflammatory and oxidative stress processes inside neuronal cells. 

This work is devoted to the study of the ability of a new Kunitz-type peptide from *H. crispa* as well as three known ones to protect neuronal cells from the actions of neurotoxins, which are the cause of PD, as well as to the search for possible mechanisms of their protective action.

## 2. Results

### 2.1. Expression and Purification of the Peptides

**Figure 1:**
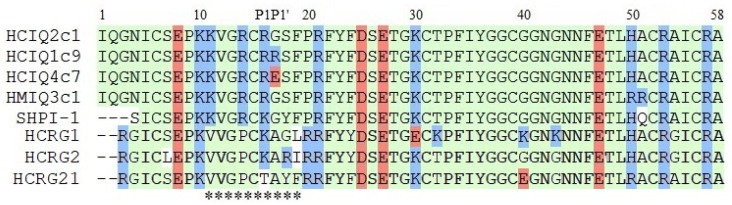
The alignment of sequences of peptides: HCIQ2c1 (UniProtKB ID: A0A6B7FBD3), HCIQ1c9 (A0A6B7FEJ3), HCIQ4c7 (A0A6B7FA07), HCRG1 (C0HJU6), HCRG2 (C0HJU7), and HCRG21 (P0DL86) from *H. crispa*; HMIQ3c1 (A0A3G2FQK2) from *H. magnifica*; and SHPI-1 (P31713) from *Stichodactyla helianthus*. P1-P1’—residues of the reactive sites of Kunitz-type peptides. The asterisks (*) below the sequences indicate the contact sites with proteases. Neutral, positively charged, and negatively charged residues are colored in green, blue, and red, respectively; non-similar residues are shown on white background.

To study the neuroprotective activity, four peptides differing from each other by the presence of charged residues were chosen from combinatorial libraries of Kunitz-type peptides of the sea anemones *H. crispa* (HCIQ2c1, HCIQ4c7, and HCIQ1c9) and *H. magnifica* (HMIQ3c1) (Figure 1).

**Figure 2:**
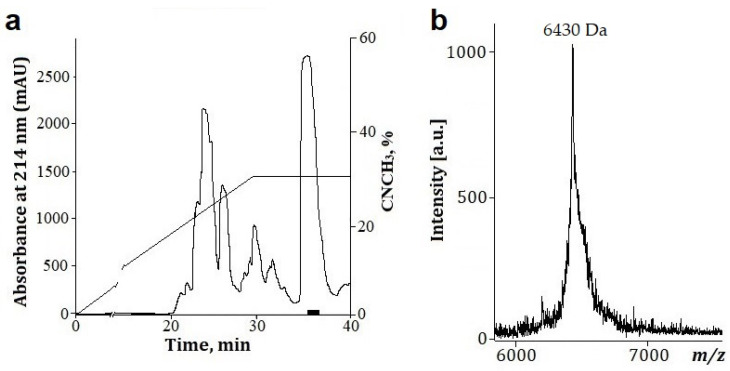
Isolation and molecular mass of HCIQ1c9: (**a**) HPLC elution profile of HCIQ1c9 on a Jupiter C4 reverse-phase column (10 × 250 mm), using a linear gradient of acetonitrile concentrations (0–70%) over 70 min with 0.1% TFA and a flow rate of 1.5 ml/min; (**b**) MALDI-TOF MS spectrum of HCIQ1c9 after RP-HPLC.

The peptides HCIQ2c1, HCIQ4c7, and HMIQ3c1 were obtained as a result of their production in *Escherichia coli,* as previously described [43,51]. This technique was successfully used to obtain HCIQ1c9. The fusion proteins expressed in *E. coli* and purified by metal affinity chromatography had an expected molecular mass of about 23 kDa and were cleaved by CNBr. Targeted peptides were purified via RP-HPLC. The retention time of HCIQ1c9 on a reverse-phase column was 35.5 min (Figure 2a), which was close to the retention times of the previously obtained peptides [43,51]. The final yield of HCIQ1c9 was 9.02 mg/L of cell culture. The molecular masses of the peptides corresponded to the expected ones, while the molecular mass of HCIQ1c9 was 6429 Da (Figure 2b). The N-terminal sequences (15 a.a.) determined by the automated Edman degradation matched well with sequences deduced from cDNA.

To determine whether the obtained recombinant peptides had spatial structures, ^1^H NMR spectroscopy was used. According to the ^1^H NMR spectra, all peptides had pronounced spatial structures, as evidenced by the wide chemical shift dispersion of amide hydrogens to the field of 8–10 ppm and the presence of resonance signals below 0 ppm (Figure S1). Narrow signal lines may indicate that the peptide structures are stable, while the equal line width shows their homogeneity.

### 2.2. Calculation of the Peptides’ Secondary Structures 

**Figure 3:**
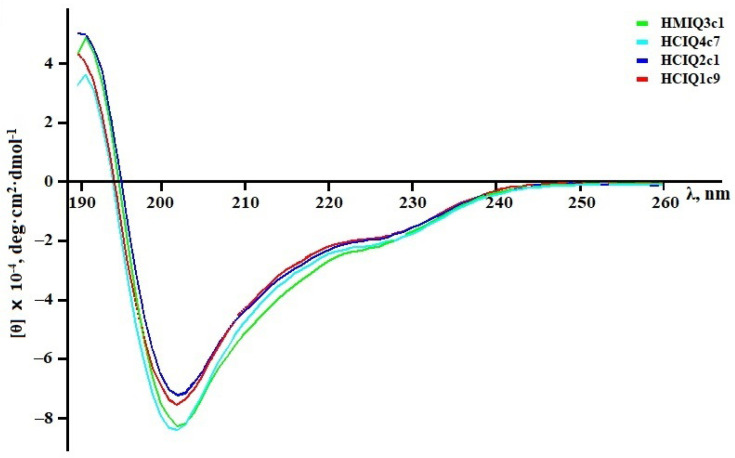
CD spectra of the peptides in deionized water in the far UV region.

To calculate secondary structural elements of peptides, circular dichroism (CD) spectroscopy was applied. CD spectroscopy is a fast, well-established, and widely used analytical technique to study secondary structures of proteins and their changes in different environments [52]. According to Figure 3, all spectra in the far UV region (190–240 nm) have similar profiles and are characterized by a positive maximum peak at 193 nm and negative minimum peaks at 202 and 225 nm. The peaks at 193 and 225 nm indicate the presence of α-helixes and β-strands, respectively, while the small magnitude of the 225 nm peak and contribution of the signal at 215 nm suggest that the peptides are partially folded. Indeed, analysis of the spectra using the Provencher–Glockner method revealed that all peptides contain both α-helixes and β-strands and an unordered structure. The peptides have an approximately equal content of secondary structural elements, while a slight increase in α-helixes and decrease in β-structure were found in HCIQ1c9 (Table 1), which might be reflected in the spatial structure and biological activity of the peptide. A comparison of peptide structures with the Kunitz-type peptides InhVJ from *H. crispa* [44] and SHPI-1 from *S. helianthus* [53], revealed similar values of secondary structural elements.

### 2.3. Trypsin-Inhibitory Constant Determination

As mentioned above, the main function of Kunitz-type peptides is the inhibition of proteases, in particular trypsin. To confirm the bioactive conformation of HCIQ1c9, its ability to inhibit trypsin was determined. Like other Kunitz-type peptides in sea anemones, HCIQ1c9 inhibited trypsin with an inhibitory constant (*K_i_*) value 6.3 × 10^−7^ M. This is similar to HCIQ4c7 (1.9 × 10^−7^ M) but one order higher than for HCIQ2c1 (5.2 × 10^−8^ M) [43] and HMIQ3c1 (5.0 × 10^−8^ M) [51]. The decrease in trypsin-inhibitory activity of HCIQ1c9 and HCIQ4c7 in comparison with HCIQ2c1 is probably associated with the substitution of Gly17 to Arg or Glu, respectively, at the P1’ position (Figure 1), which is an important residue for serine protease inhibition [55].

### 2.4. Modeling of Peptide Complexes with Trypsin

To determine the residues that affect the nature of the interaction with trypsin, the 3D models of peptides and their complexes with bovine trypsin were generated. A 1.71 Å resolution X-ray structure of the bovine trypsin complex with SHPI-1 (PDB ID: 3M7Q [56]) was exploited as a template, since SHPI-1 is a close homolog of the peptides sharing 91 to 93% identity (Figure 1). The conformational analysis models revealed that 90–98% of the residues occupy the most favored regions of the Ramachandran plot, with no steric hindrance, reflecting sufficient model quality.

**Figure 4:**
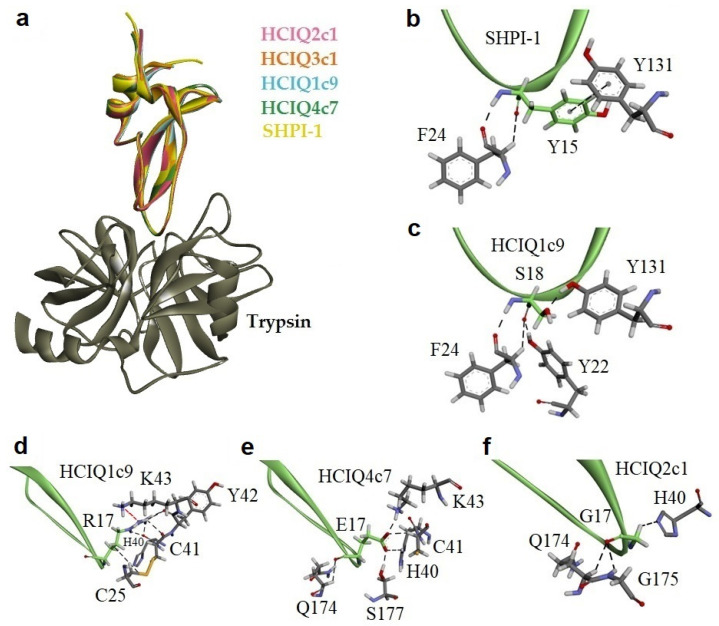
Comparative models of the peptide complexes with trypsin. (**a**) Ribbon representation of peptide complexes. Intermolecular interactions of residues at positions P1’ (**b**,**c**) and P2’ (**d**–**f**) with trypsin molecule. Residues are presented as sticks; hydrogen, oxygen, nitrogen, and sulfur atoms are colored in white, red, blue, and orange, respectively. Interactions are shown as dotted lines, unfavorable interactions between Arg17 of HCIQ1c9 and Lys43 of trypsin are shown in red.

Despite point replacements between the peptides themselves and SHPI-1, the overall structures and interfaces of all obtained complexes were almost identical to the prototypical trypsin complex with SHPI-1 (Figure 4a). The positively charged residue Lys13/Arg16 (SHPI-1/studied peptides) at position P1 makes salt bridges and H-bonds with the Asp171 at site 1 in trypsin, while Arg11/Arg14 at position P3 interacts with site 3 residues (Asn79, Thr80, Gln155, Trp 193, and Gly194). The interaction with the catalytic pocket is a characteristic if trypsin complexes with Kunitz-type inhibitors, whereby the complex stabilization by the P3 residue is substantially enhanced for sea anemone peptides versus BPTI or APPI because of Arg to Pro substitution [56]. The interfaces of HCIQ2c1 or HMIQ3c1 are distinguished from SHPI-1 due to the presence of Ser18 instead of Tyr. According to the obtained results, Tyr15 from both SHPI-1 and Ser18 in peptides could make contacts with neighboring trypsin residues Tyr22, Phe24, and Tyr131, indicating their similar roles in complex formation (Figure 4b,c). The peptides HCIQ1c9 and HCIQ4c7 have oppositely charged residues Arg17 and Glu17 at position P1’, respectively. Both Arg17 in HCIQ1c9 and Glu17 in HCIQ4c7 form an H-bond network with trypsin, but Glu17 makes salt bridges with Lys43, while the unfavorable closeness of Arg17 and Lys43 to each other lead to these residues being pushed off (unfavorable interaction) (Figure 4d–f).

### 2.5. Temperature Effects on the Secondary Structure and Biological Activity of Peptides

**Figure 5:**
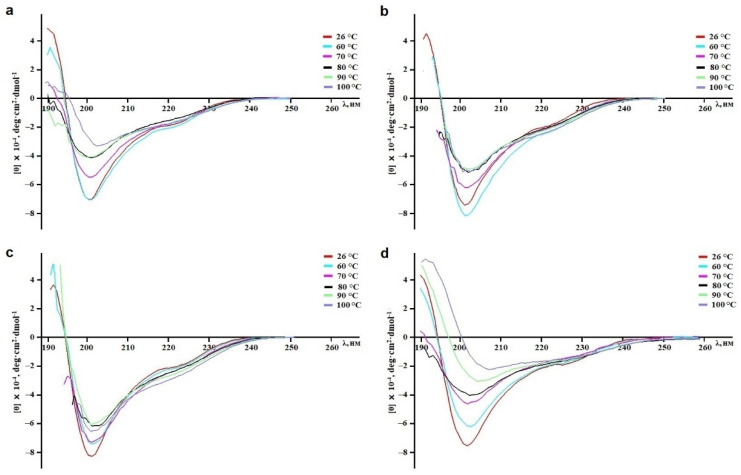
Evaluation of the thermal stability of peptides in deionized water via CD spectroscopy. Recordings of the CD spectra of HCIQ2c1 (**a**), HMIQ3c1 (**b**), HCIQ4c7 (**c**), and HCIQ1c9 (**d**) were conducted in the far UV region (190–240 nm) after the solutions were incubated over a temperature range from 25 °C to 100 °C for 20–25 min.

**Figure 6:**
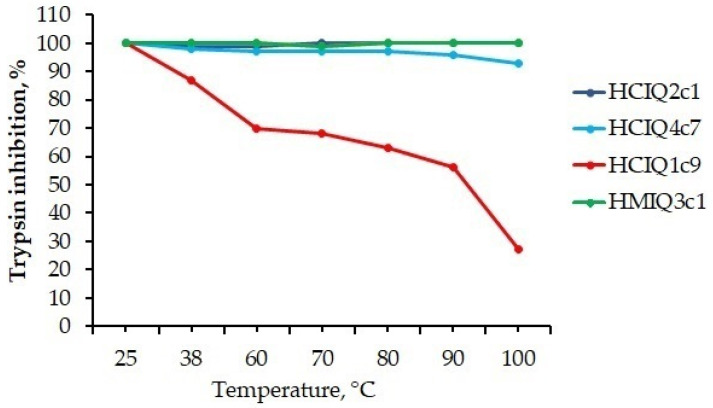
The effects of temperature on the trypsin-inhibitory activity levels of peptides. Peptides were incubated at the temperatures for 30 min and their residual activities were measured.

The temperature effects on the secondary structure and biological activity of peptides were analyzed using both CD spectroscopy and a protease-inhibitory assay. The CD spectra of HCIQ2c1 and HCIQ4c7 as well as of HMIQ3c1 and HCIQ1c9 solutions showed no significant changes after heating until 90 °C and 80 °C, respectively (Figure 5). Further heating of the solutions to 100 °C resulted in decreased positive peaks at 193 nm in HCIQ2c1, HCIQ4c7, and HMIQ3c1 spectra, as well as decreased spectral peaks at 202 and 225 nm in the HCIQ1c9 spectrum, indicating a reduction in α-helixes in the former and the initiation of protein unfolding in the latter. These data were confirmed by the trypsin-inhibitory activity results (Figure 6). HCIQ2c1 and HMIQ3c1 were shown to inhibit trypsin completely at all temperature ranges, including at 100 °C, while HCIQ4c7 activity was weakly decreased (by 7%) after heating to 100 °C. Regarding HCIQ1c9, the heating of the solution in the range from 60 °C to 100 °C resulted in decreases in inhibitory activity of 30–70%. Thus, the obtained results indicated that HCIQ2c1, HMIQ3c1, and HCIQ4c7 have high thermostability, retaining their conformation on the secondary structural level and their biological activity until 90–100 °C.

### 2.6. The Influence of the Peptides on 6-OHDA-, Paraquat-, Rotenone-, and MPP^+^-Induced Toxicity

**Figure 7:**
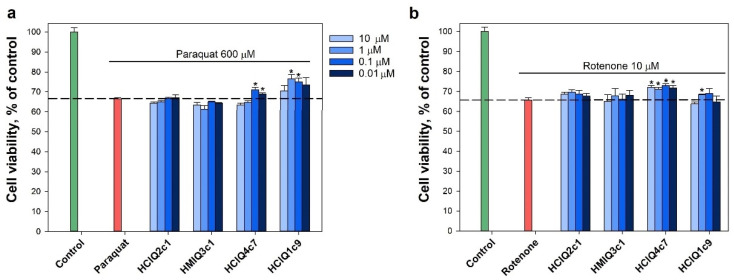
Effects of the peptides on neuroblastoma cell viability in the presence of 600 μM paraquat (**a**) and 10 μM rotenone (**b**). The data are shown as means ± SE; * —*p* < 0.05.

The peptides were tested for cell viability in in vitro 6-OHDA-, rotenone-, paraquat-, and MPP^+^-induced Parkinson’s disease models. All peptides were non-toxic on Neuro-2a cells at a concentrations up to 10 µM (Figure S2). The viability of neurotoxin-treated cells was about 65% versus control (Figure 7). The peptide HCIQ1c9 at concentrations from 0.1 to 10 μM did not influence the viability of 6-OHDA-treated cells in contrast to HCIQ4c7 and HCIQ2c1, increasing the cell viability in this model by 14% and 47%, respectively [43]. The peptides HCIQ2c1 and HMIQ3c1 at concentrations up to 10 μM did not influence the viability of paraquat-treated cells, while HCIQ4c7 and HCIQ1c9 increased the cells viability in a dose-dependent manner, with the maximum activity levels of up to 6.7 ± 1.7% and 14.8 ± 3.3% at concentration of 1 and 0.1 μM, respectively (Figure 7a). Moreover, HCIQ4c7 and HCIQ1c9 increased the viability of rotenone-treated cells in a statistically significant manner (Figure 7b). The effect of HCIQ4c7 on the cell viability did not depend on its concentration and reached 10.8 ± 1.4% at 0.1 μM, while HCIQ1c9 increased the cell viability by up to 4.0 ± 0.6% at 1 μM. Regarding the MPP^+^-induced toxicity model, HCIQ1c9 only revealed a cytoprotective effect; the peptide at a concentration of 10 μM increased the cell viability by up to 9.97 ± 2.9%.

### 2.7. Effect of Peptides on Paraquat- and Rotenone-Induced ROS Formation

**Figure 8:**
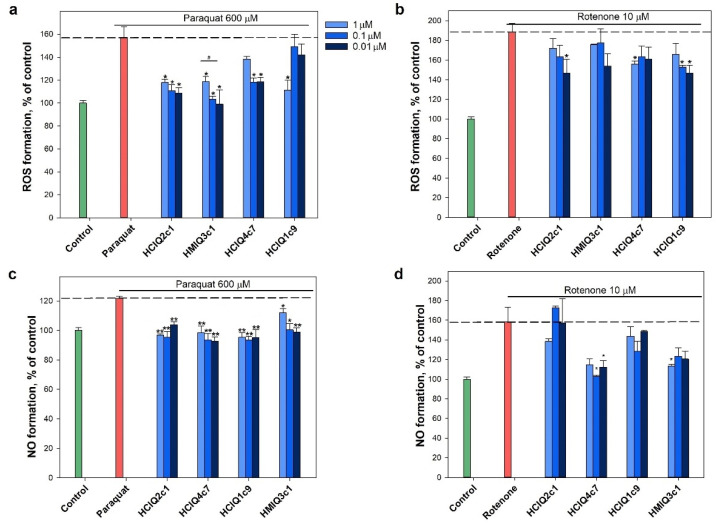
Effects of the peptides on intracellular ROS and NO formation. The effects of the peptides on ROS formation into Neuro-2a cells treated by 600 μM paraquat (**a**) and 10 μM rotenone (**b**). The effects of peptides on NO formation into the cells treated by 600 μM paraquat (**c**) and 10 μM rotenone (**d**). Cells were incubated with peptides for 1 h at 37 °C, then 3 or 1 h with paraquat or rotenone, respectively. The data are shown as the means ± SE; *—*p* < 0.05, **—*p* < 0.01, #—*p* < 0.05 when comparing concentrations of the same peptide.

The peptides inhibited both rotenone- and paraquat-induced ROS formation (Figure 8a,b). The decrease in ROS levels caused by the peptides in the paraquat-induced cytotoxicity model was stronger than in the presence of rotenone. A reliable dose-dependent effect in the presence of paraquat was revealed for HMIQ3c1, with a maximum decrease in ROS levels (less than control level) of 36.9 ± 7.9% at a concentration of 0.01 μM. HCIQ2c1 and HCIQ4c7 at the same concentrations decreased ROS levels by 30.7 ± 1.8% and 24.6 ± 2.5%, respectively, while HCIQ1c9 inhibited ROS levels by 29.3 ± 5.6% at a concentration of 1 μM, a statistically significant result (Figure 8a). In the presence of rotenone, statistically significant decreases in ROS levels were revealed for HCIQ2c1 and HCIQ1c9 (by 22.0 ± 7.3% and 22.0 ± 4.2%, respectively) at a concentration of 0.01 μM, HCIQ4c7 inhibited ROS by 17.3 ± 1.7% at a concentration of 1 μM (Figure 8b). In addition, all peptides decreased NO formation induced by paraquat in a statistically significant manner (Figure 8c). HCIQ4c7 at a concentration of 0.01 μM showed a maximal effect, decreasing NO by 22.3 ± 2.4%, which was less than the control level. In the rotenone-induced toxicity model, the peptides (except HCIQ2c1) significantly decreased NO, while HCIQ4c7 at a concentration of 0.1 μM decreased NO to the control level (by 20.5 ± 0.1%), which was a statistically significant result (Figure 8d). 

### 2.8. Free Radical Scavenging of the Peptides

**Figure 9:**
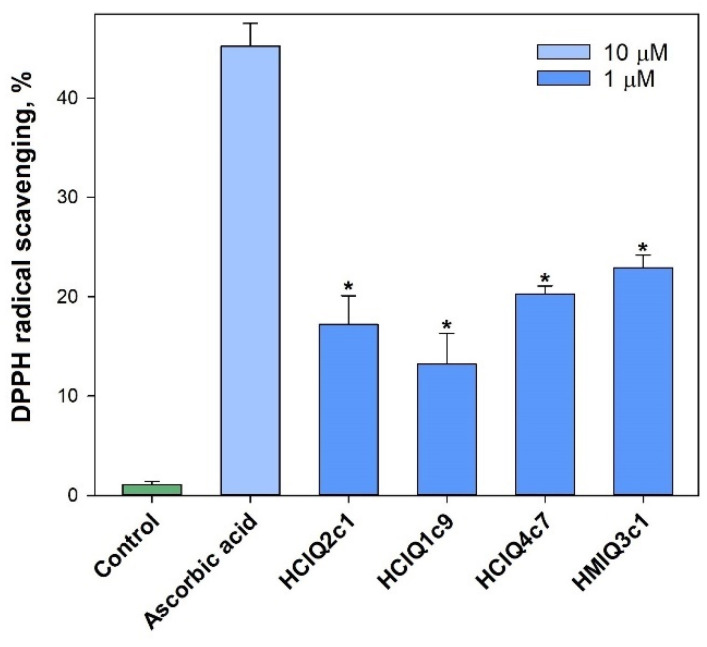
Scavenging activity levels of the peptides against DPPH radicals outside the cells. The data are shown as the mean ± SE; *—*p* < 0.05.

The studied peptides were tested for their ability to scavenge radicals using a DPPH radical scavenging cell-free assay. The obtained results revealed that all peptides reduced the DPPH radicals (Figure 9). The maximum antiradical effect was shown for HMIQ3c1, which scavenged 22.9 ± 1.3% of DPPH radicals.

### 2.9. Effects of the Peptides on TRPV1 Channels

**Figure 10:**
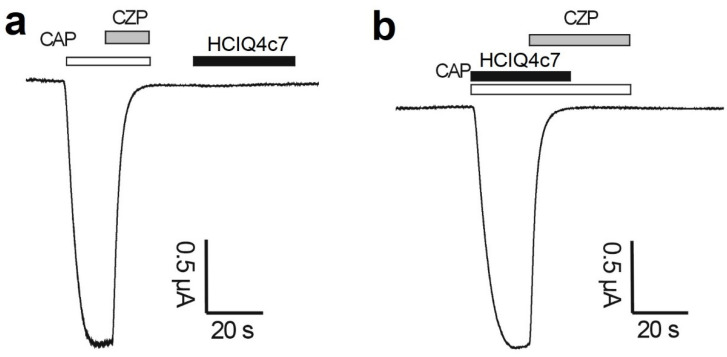
Effect of HCIQ4c7 on TRPV1 channels expressed in *X. laevis* oocytes. Whole-cell current traces of capsaicin (CAP = 2 µM) and capsazepine (CZP = 10 µM). Applications of HCIQ4c7 alone (**a**) or in the presence of capsaicin (**b**) are shown. The image was taken using pClamp Clampfit 10.0 (Molecular Devices, Downingtown, PA, USA) and Origin 7.5 software (Originlab, Northampton, MA, USA).

The electrophysiological study of the peptides was carried out on TRPV1 channels expressed in *Xenopus laevis* oocytes. The activation of the TRPV1 channels was initiated via the application of 2 µM capsaicin (CAP). The application of 10 µM capsazepine (CZP) resulted in blocking of the channels. It was found that the peptides at a concentration of 10 μM did not exert any effects on TRPV1 channels, either when the peptides were administered alone (Figure 10a) or during co-application together with capsaicin (Figure 10b).

### 2.10. Effects of the Peptides on ATP-Induced Neuro-2a Cell Death

**Figure 11:**
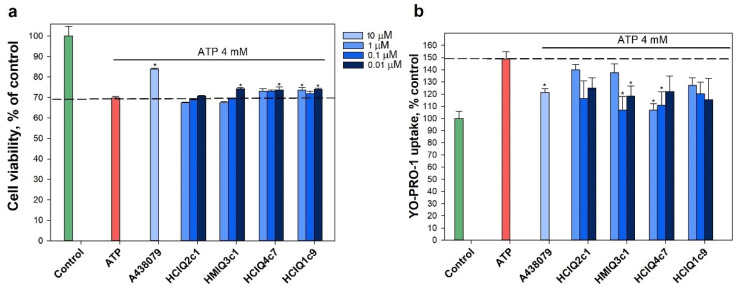
The effects of the peptides on ATP-induced Neuro-2a cell viability and activation of P2X7R. (**a**) Impact of the peptides on Neuro-2a cell viability in the presence of 4 mM ATP. The peptides were tested at concentrations of 0.01, 0.1, and 1.0 μM. Cells were incubated with peptides for 1 h at 37 °C, then for 48 h with ATP. (**b**) Effects of the peptides (0.01, 0.1, and 1.0 μM) on YO-PRO-1 uptake in Neuro-2a cells. ATP was used as a P2X7R agonist. A438079 (10 μM) was used as a standard inhibitor of P2X7R. The peptide effects were evaluated against the effects of ATP. The data are shown as means ± SE; *—*p* < 0.05 compared to the effect of ATP alone.

To establish whether 4 mM ATP leads to the death of Neuro-2a neuroblastoma cells, the cells were incubated with or without ATP for 48 h, and the percentage of viable cells was then determined via MTT assay. Incubation with 4 mM ATP led to a significantly increased percentage of total dead cells (up to 30.15 ± 0.68%) compared with that in control cells (Figure 11a). To determine whether ATP-induced Neuro-2a cell death is caused by P2X7R activation, the cells were pre-incubated with or without 10 μM A438079, which is the inhibitor of P2X7R [57], and then were incubated in the absence or presence of 4 mM ATP for 48 h. Pre-incubation of cells with 10 μM A438079 increased the cell viability by 21.3 ± 0.5% compared to the cells incubated with ATP alone, indicating that this process is partially mediated by P2X7R activation. The peptides (except HCIQ2c1) had a slightly protective effect on ATP-induced cells. Statistically significant increases in cell viability were observed for HCIQ4c7 (0.1 μM), HCIQ1c9 (0.01 μM), and HMIQ3c1 (0.01 μM), amounting to 4.7 ± 0.74%, 5.96 ± 0.72%, and 6.46 ± 0.65%, respectively, as compared to cells incubated with ATP. Nevertheless, the dependence of the cytoprotective properties of peptides on the studied concentrations is not clearly shown.

ATP (4 mM) increased the YO-PRO-1 dye penetration into Neuro-2a cells by 49.4 ± 5.9% (Figure 11b). HMIQ3c1 and HCIQ4c7 at concentrations of 0.1 μM and 1 μM significantly reduced ATP-induced dye uptake by 28.1 ± 7.5 and 28.5 ± 3.7%, respectively, showing superior results to A438079 (18.5 ± 3.2%). HCIQ1c9 and HCIQ2c1 also inhibited ATP-induced dye uptake, but their effects were not statistically significant. Therefore, HCIQ4c7 and HMIQ3c1 were selected for further study.

### 2.11. Effects of the Peptides on ATP-Induced Ca^2+^ Influx into Neuro-2a

**Figure 12:**
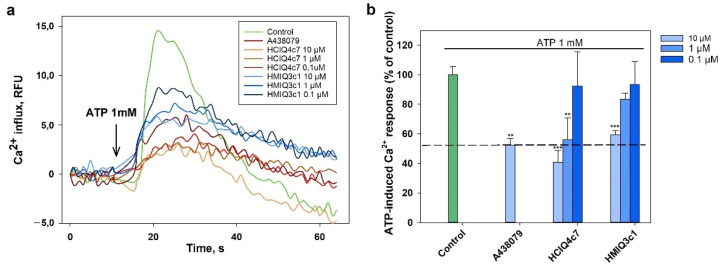
Influence of HCIQ4c7 and HMIQ3c1 on ATP-induced calcium influx in Neuro-2a cells. (**a**) Representative time course of [Ca^2+^]_I_ increases induced by ATP (1 mM) alone or in the presence of the peptides or the P2X7R blocker A438079 (10 μM) in Neuro-2a cells, as measured with Fluo-8. (**b**) Effect of pre-incubation of Neuro-2a cells with the peptides (0.1, 1.0, and 10.0 μM) or A438079 (10 μM) on Ca^2+^ influx caused by ATP (1 mM). The data are shown as the means ± SE; **—*p* < 0.01, and ***—*p* < 0.001 compared to the effect of ATP alone.

To determine whether HCIQ4c7 and HMIQ3c1 inhibit the activation of P2X7R, the Ca^2+^ influx induced by channel gating (pore expansion) was measured. Neuro-2a cells were loaded with the Ca^2+^-selective fluorescent probe Fluo-8, then cellular calcium responses were recorded. A large sustained increase in [Ca^2+^]_i_ was recorded after the addition of 1 mM ATP, indicating that it was also mediated through the purinergic receptor. This increase was significantly reduced in the presence of 10 μM A438079 (Figure 12a). The peptides reduced the Ca^2+^ response at all tested concentrations (Figure 12b). A438079 decreased the calcium response by 47.7 ± 4.4% relative to the control level, while HCIQ4c7 and HMIQ3c1 at a concentration of 10 μM decreased the stable calcium response by 59.1 ± 7.8% and 40.6 ± 2.6%, respectively. These data indicate that the peptides are capable of affecting the P2X7R activation.

### 2.12. Interaction of the Peptides with P2X7R Subunit

**Figure 13:**
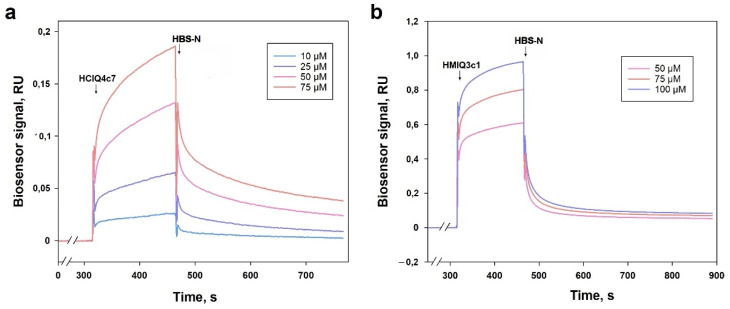
Binding sensorgrams of immobilized P2X7R with HCIQ4c7 (**a**) and HMIQ3c1 (**b**) at 25 °C. Correspondence of line colors to the peptide concentrations is shown in frame. The injection times of the peptides and HEPES-buffered saline (HBS-N) are shown by a black arrow.

To reveal that HCIQ4c7 and HMIQ3c1 influence P2X7R, their interactions with P2X7R were estimated using the surface plasmon resonance (SPR) method. For this purpose, 12.3 ng/mm^2^ human recombinant P2X7R was immobilized on a Biacore 3000 optical biosensor chip. The direct binding of the peptides with P2X7R was detected and *K_d_* values for the complexes of P2X7R with HCIQ4c7 and HMIQ3c1 were calculated as 45.5 and 43.3 μM, respectively. The dose–response curves from the SPR kinetic analysis are shown in Figure 13.

## 3. Discussion

Neurodegenerative disorders are socially significant diseases; they are some of the most common diseases globally among the elderly, and their frequency is steadily increasing. To date, there is growing evidence that in the development of neurodegenerative diseases, along with the degeneration of dopaminergic neurons (such as in PD), oxidative stress and inflammation play a significant role [5]. The focus of research in studying the etiology of these diseases is shifting to new targets, such as ion channels and enzymes. In this regard, the creation of drugs based on natural compounds with anti-inflammatory or antioxidant activity, specifically interacting with ion channels, enzymes, and other targets involved in neurodegenerative disorders, is vital for developing strategies for their treatment.

The peptides HCIQ2c1and HCIQ4c7 from *H. crispa* and HMIQ3c1 from *H. magnifica* interact with several serine proteinases, including inflammatory ones, and they were also described as the first Kunitz-type peptides with neuroprotective activity [43,51,58]. A new peptide, HCIQ1c9, with Gly17Arg at position P1’ of the reactive site was also found in a combinatorial library of *H. crispa* Kunitz-type peptides [43] and produced in *E. coli*. Thus, in this work four isoforms of Kunitz-type peptides with point a.a. substitutions were studied in various in vitro models.

Analysis of the superposition of 3D models of the peptides studied here and of SHPI-1 from *S. helianthus* indicated that the peptides have a spatial structure intrinsic of Kunitz folding. Despite the single replacements, the interfaces of the studied peptides and SHPI-1 are almost identical to SHPI-1. According to CD spectra, all peptides contain both α-helixes and β-strands comparable with SHPI-1. 

The active spatial conformation of the peptides was also confirmed by the presence of trypsin-inhibitory activity. As shown previously, HCIQ2c1 and HMIQ3c1 inhibited trypsin at 10^−8^ M and HCIQ4c7 (Gly17Glu) at 10^−7^ M. The peptide HCIQ1c9 (Gly17Arg) also inhibited trypsin, with a *K_i_* value (6.3 × 10^−7^ M) close to HCIQ4c7. Thus, both negatively and positively charged residues at position P1’ led to decreases in the trypsin-inhibitory activity of the peptides, with HCIQ4c7 beings a stronger trypsin inhibitor than HCIQ1c9. This was due to the residue Glu17, which can make additional contact with Lys43 in trypsin, while Arg17 from HCIQ1c9 experiences an unfavorable interaction with the trypsin residue.

One of the important characteristics of Kunitz-peptides is the strong stability of the domain structure thanks to the presence of three disulfide bonds. This feature allows them to maintain their protease-inhibitory activity over a wide range of temperatures and pH values. Indeed, the Kunitz peptide SdPI from the scorpion *Lychas mucronatus* [58] and BmTI-A from tick *Rhipicephalus microplus* [59] were shown to be thermostable, while BmSPI51 from the cocoon of the silkworm *Bombyx mori* is both heat- and pH-resistant [60]. Based on the CD spectra and residual trypsin activity levels, we revealed that HCIQ2c1, HCIQ4c7, and HMIQ3c1 are thermostable peptides retaining the conformation of the active molecules up to 90–100 °C, whereas the trypsin-inhibitory activity of HCIQ1c9 is reduced by up to 70% in the temperature range of 60–100 °C, an observation that was confirmed by the CD spectrum. 

Through our study, we demonstrated that the substitution of the residues at position P1’ of the peptides contributes to their biological activity. Earlier, we revealed that HCIQ2c1 has stronger neuroblastoma cell viability against 6-OHDA than HCIQ4c7 with Gly17Glu substitution [43], while HMIQ3c1 with His50Arg and Ala51Arg substitutions did not affect 6-OHDA-induced cell toxicity [51]. Here, HCIQ1c9 showed a maximum protective effect in the paraquat-induced cell death model, while HCIQ4c7 was the best in the rotenone-induced cytotoxicity model. Furthermore, HCIQ1c9 exhibited protective effects on cells in the presence of MPP^+^. Both rotenone and paraquat are known to induce oxidative stress in neuronal cells. Rotenone promotes mitochondrial complex I inhibition, resulting in an increase in ROS production, while paraquat induces redox cycling in the cytosol, altering the mitochondrial function indirectly [12]. The peptides inhibited ROS formation in the presence of both paraquat and rotenone. It was suggested that the inhibition of ROS formation is associated with the antiradical activity of the peptides. Indeed, these peptides interacted with free radicals, which may be one of the reasons for the reduced ROS levels. On the other hand, it is known that ion channels such as Kv and TRP and cell receptors such as P2X7 are actively involved in the regulation of neuronal processes, including inflammation, and are considered potential therapeutic targets for PD [61,62,63]. Recently, we showed that Kunitz-type peptides, namely HCRG1 and HCRG2, which are blockers of Kv channels, maintained ROS production in 6-OHDA-treated cells at the reference level [50]. Moreover, HCRG21, a blocker of TRPV1, showed significant neuroprotective action, decreasing ROS production below the control (up to 87%) [44]. The studied peptides were not active in either Kv [43] or TRPV1 channels, despite the high sequence homology with HCRG1, HCRG2, and HCRG21.

Cell death induced by oxidative stress is accompanied by the release of ATP, which at high concentrations activates P2X7R-associated channel opening, leading to osmotic swelling and ultimately to cell death [22,23]. Therefore, the application of a P2X7R inhibitor can attenuate the progress of a neurodegenerative process. The studied peptides were found to inhibit the uptake of YO-PRO-1 staining, which is able to penetrate through the ion channels formed by P2X7R in Neuro-2a cells [22,64]. HMIQ3c1 and HCIQ4c7 demonstrated the best effects, almost completely inhibiting the ATP-induced YO-PRO-1 uptake. It should be noted, that both HMIQ3c1 and HCIQ4c7 at concentrations of 0.1 and 1 μM, respectively, more effectively inhibited the dye penetration than 10 μM A438079, a selective inhibitor of P2X7R. The peptide effect on P2X7R activation was confirmed by the results of Ca^2+^ influx measurements and SPR. Upon binding to ATP, P2X7Rs act as nonselective cation channels, resulting in a steady calcium influx response and increasing the intracellular concentration of calcium ([Ca^2+^]_i_). The obtained results showed that both peptides reduced Ca^2+^ influx and the Ca^2+^ response into Neuro-2a cells, with HCIQ4c7 more effectively decreasing the calcium response than A438079. Moreover, HCIQ4c7 and HMIQ3c1 bound to the P2X7R extracellular domain with similar *K_d_* values, indicating the formation of stable complexes and excluding non-specific binding. This is direct evidence of the ability of the peptides to bind to the studied target, which may partially explain their neuroprotective activity in PD models.

One of the important requirements for potential neuroprotective compounds is their ability to pass through the blood–brain barrier (BBB), which restricts the entry of proteins and undesirable substances to cerebral tissues. Because most drugs do not cross the BBB, few treatments are available against neurodegenerative diseases, including PD. 

It has been shown that the Kunitz domain in the β-amyloid precursor protein and Kunitz-type peptide from the bovine pancreas, BPTI, effectively crosses the BBB via low-density lipoprotein receptor-related protein (LRP) [65,66]. Moreover, short peptides create the basis for the BPTI sequence, which has been demonstrated to effectively cross the BBB [66]. The studied peptides have the same folds, including the pattern responsible for penetration through the BBB. 

On the other hand, the BBB is known to become more permeable during the inflammatory process. As mentioned above, P2X7R is actively involved in the inflammatory process. The receptor associated with pannexin-1 and the P2X4 receptor are able to stimulate the ROS production induced by ATP, which leads to the activation of NLRP3 (NOD-, LRR-, and pyrin domain-containing protein 3) inflammasome and further release of proinflammatory cytokines IL-1β and IL-18 [67]. The inflammation carried out by IL-1β is found to promote the dysfunction and hyperpermeability of the BBB [68,69]. Taken together, the data allow us to assume that the studied peptides might be able to pass through the BBB, at least during inflammation.

Thus, the studied peptides are able to protect neuronal cells against PD inductors via the inhibition of ROS formation and ATP-induced P2X7R activation. However, the molecular mechanism of their interaction with P2X7R and passage through the BBB remains uncertain and requires further detailed study.

## 4. Materials and Methods

### 4.1. Expression and Isolation of Kunitz-Type Peptides

The pET32b(+)/*hciq1c9* construction was synthesized by JSC Eurogen (Russia). The Kunitz-type peptides HCIQ2c1, HCIQ4c7, and HMIQ3c1, as well as a new peptide HCIQ1c9, were obtained as described in [43,51]. The recombinant plasmids based on pET32b(+) and carrying the genes of the target peptides fused with thioredoxin were transformed into *E. coli*. The cells were cultured at 37 °C in Luria–Bertani medium containing 100 μg/mL ampicillin until reaching the optical density (OD_600_) of ~0.5. After induction with IPTG across a concentration range of 0.2–0.5 mM, the cells were incubated at 37 °C for 3 h. The presence of recombinant peptides was determined in 12% polyacrylamide gel by Laemmli’s SDS-PAGE method [70]. Cell precipitates were resuspended in the starting buffer (400 mM NaCl, 20 mM Tris-HCl buffer, pH 8.0) and ultrasonicated on ice. Fusion proteins were purified under native conditions on a Ni-NTA agarose (Qiagen, Venlo, The Netherlands) according to the manufacturer’s instructions and cleaved using CNBr overnight at room temperature with a CNBr-to-protein molar ratio of 600:1 [71]. The recombinant peptides were purified from reaction mixture on a Jupiter C4 10 × 250 mm reverse-phase column (Phenomenex, Torrance, CA, USA) using a linear gradient of acetonitrile (from 0% to 70%) with 0.1% trifluoroacetic acid (TFA) over 70 min with a constant flow rate of 1.5 mL/min. 

### 4.2. N-Terminal Amino Acid Sequence Analysis

HCIQ1c9 was treated using 6 M guanidine hydrochloride in 0.5 M Tris-HCI buffer, pH 8.5, containing 2 mM EDTA. Then, dithiothreitol was added and the mixture was incubated for 4 h at 40 °C. Thiol groups of cysteine residues were modified by 50% 4-vinylpyridine in isopropanol for 20 min at room temperature in the dark. The reaction mixture was separated on a Nucleosil C18 4.6 × 250 mm reverse-phase column (Phenomenex, Torrance, CA, USA) using a concentration gradient of acetonitrile (from 0% to 70%) with 0.1% TFA in 160 min, with a constant flow rate of 0.5 mL/min [49]. The N-terminal sequence of HCIQ1c9 (15 a.a.) was determined in duplicate via automatic Edman degradation on a Procise 492c LC sequencer (Applied Biosystems, Bedford, MA, USA) equipped with a Series 200 UV–Vis detector (Perkin Elmer, Waltham, MA, USA) for analysis of phenylthiohydantoyl amino acid derivatives according to the supplier’s instructions.

### 4.3. MALDI-TOF MS Analysis 

MALDI-TOF MS spectra of peptides were recorded using an Ultra Flex III MALDI-TOF/TOF mass spectrometer (Bruker, Bremen, Germany) with a nitrogen laser SmartBeam (355 nm), reflector, and potential LIFT tandem modes of operation. Sinapinic acid was used as the matrix. External calibration was employed using a peptide InhVJ with *m*/*z* 6107 [72] and its double-charged variant at *m*/*z* 3053.

### 4.4. One-Dimensional NMR Spectroscopy

The ^1^H NMR spectra of peptides were acquired at 30 °C on a Bruker Avance III 700 MHz spectrometer (Bruker Biospin, Billerica, MA, USA) equipped with a triple-resonance z-gradient TXO probe. Peptides were dissolved in 90% H_2_O/10% D_2_O (Deutero GmbH, Kastellaun, Germany) over a concentration range of 1.5–2 mg/mL. Excitation sculpting with gradients [73] was applied to suppress strong solvent resonance, while the chemical shift of their signals was arbitrarily chosen as 4.7 ppm. TopSpin 3.6 (Bruker Biospin, Billerica, MA, USA) was used for the acquisition and processing of the spectra.

### 4.5. Circular Dichroism Spectroscopy

Circular dichroism (CD) spectra were recorded on a Chirascan-Plus CD spectropolarimeter (Applied Photophysics, Leatherhead, UK) in quartz cuvettes with an optical path length of 0.1 cm for the peptide spectrum region. The peptides were dissolved in deionized water (40 ng/mL) and incubated at temperatures ranging from 25 °C to 100 °C for 20–25 min before recording the CD spectra. The secondary structure elements were calculated using the Provencher–Glockner method [74] using advanced Provencher calculation programs from the CDPro software package (Leatherhead, UK) [75].

### 4.6. Trypsin-Inhibitory Activity

The trypsin-inhibitory activity of HCIQ1c9 was estimated according to the standard procedure using N-α-benzoyl-D,L-arginine p-nitroanilide (BAPNA) (Sigma-Aldrich, St. Louis, MO, USA) as a substrate. The trypsin inhibition constant was determined using Dyxon’s method [76] using substrate concentrations of 0.6 and 1.2 mM. The trypsin concentration was 208 nM. The range of peptide concentrations was 0–20 μM. The constant was calculated based on the results of three parallel experiments. Computational error limits were in the range of 0.1–0.3%.

The temperature influence on the activity levels of peptides was assessed by measuring their residual inhibitory activity levels. After preincubation at temperatures ranging from 25 to 100 °C for 25 min, the peptide solutions were added to trypsin solution and the reaction mixtures were incubated for 10 min at 37 °C, then 1.2 mM BAPNA was added, followed by incubation at 37 °C for 30 min. The final concentration of the peptides was 7 μM. Substrate hydrolysis was measured at 410 nm. The residual activity was calculated based on the results of three parallel experiments according to the following equation [60]:

% = (1 − residual enzyme activity/enzyme activity without inhibitor) × 100

### 4.7. Modeling of Peptide–Trypsin Complexes

The comparative models of complex 3D structures were generated using the (PS)^2^ web server [77]. The peptide sequences HCIQ2c1 (UniProtKB ID: A0A6B7FBD3), HMIQ3c1 (A0A3G2FQK2), HCIQ1c9 (A0A6B7FEJ3), HCIQ4c7(A0A6B7FA07), and bovine cationic trypsin (P00760) were used as input data and the spatial structure of homologous peptide SHPI-1 in complex with trypsin (PDB ID: 3M7Q [56]) was chosen as the template. The obtained models were further optimized using a fragment-guided molecular dynamics (FG-MD) algorithm [78] or UCSF Chimera 1.11.2rc software [79] using the Amber ff99SB protein force field and analyzed with the QMEAN server and other model quality assessment tools available within the SWISS-MODEL workspace [80]. The residues contributing to complex interfaces were identified using Discovery Studio 4.0 Visualizer (Accelrys Software Inc., San Diego, USA), PDBePISA [81], and CONSRANK web tools [82]. Visualization was performed using Discovery Studio 4.0 Visualizer software.

### 4.8. Cell Line and Culture Conditions

The murine neuroblastoma cell line Neuro-2a was purchased from ATCC (CCL-131, American Type Culture Collection, Manassas, VA, USA). Cells were cultured in Dulbecco’s modified Eagle’s medium (DMEM) (Biolot, St. Petersburg, Russia) containing 10% fetal bovine serum (Biolot, St. Petersburg, Russia) and 1% penicillin/streptomycin (Biolot, St. Petersburg, Russia) according to ATCC’s instruction. Cells were incubated at 37 °C in a humidified atmosphere containing 5% CO_2_ (*v*/*v*). Before the experiments, Neuro-2a cells at a concentration of 1 × 10^4^ cells/well were dispensed into 96-well plates and incubated for 24 h in a humidified atmosphere containing 5% CO_2_ to allow cell attachment. 

### 4.9. Cell Viability Assay (MTT Method)

Peptide stock solutions were prepared in deionized water at a concentration of 10 mM. All tested compounds were added to the plate wells at a volume of 20 μL and diluted in PBS to final concentrations of 0.01, 0.1, 1.0, and 10.0 μM. 

Then, 20 μL of substance solution was loaded into the cells and incubated for 24 h followed by replacement of the medium with tested substances and 100 μL of fresh medium. Then, 10 μL of MTT (3-(4,5-dimethylthiazol-2-yl)-2,5- diphenyltetrazolium bromide) (Sigma-Aldrich, St. Louis, MO, USA) stock solution (5 mg/mL) was added to each well and the microplate was incubated for 4 h. Next, 100 μL of SDS-HCl solution (1 g SDS, 10 mL dH_2_O, 17 μL 6N HCl) was added to each well followed by incubation for 4–18 h. The absorbance of the converted dye formazan was measured using a Multiskan FC microplate photometer (Thermo Scientific, Waltham, MA, USA) at a wavelength of 570 nm [83,84]. All experiments were repeated in triplicate. Cytotoxic activity was expressed as the percentage of cell viability.

### 4.10. In Vitro Paraquat-, Rotenone-, MPP^+^, 6-OHDA, and ATP-Induced Cytotoxicity Assays

At the end of the preincubation period, the cells were treated with peptides at concentrations of 0.01, 0.1, 1, and 10 μM for 1 h, after which 600 μM paraquat, 10 μM rotenone, 80 μM of 6-OHDA, 1 mM MPP^+^, or 4 mM ATP (Sigma-Aldrich, St. Louis, MO, USA) was added. The cells incubated with or without inductors were used as positive and negative controls, correspondingly. Cell viability was measured after 24 h (with paraquat, rotenone and 6-OHDA) or 48 h (with ATP and MPP^+^) using the MTT assay [85,86,87]. The results are presented as a percentages, taking the cell viability when treated with the inductor as 100%.

### 4.11. ROS and NO Analyses in Paraquat- and Rotenone-Treated Cells

Neuro-2a cells were incubated with peptides at concentrations of 0.01, 0.1, and 1 µM for 1 h. Then, paraquat (600 μM) or rotenone (10 µM) was added to each well and cells were incubated for 3 or 1 h, respectively. To study the ROS formation, the 2,7-dichlorodihydrofluorescein diacetate (H2DCF-DA) assay was performed according to the manufacturer’s instructions (Molecular Probes, Eugene, OR, USA). H2DCF-DA solution was added to each well, such that the final concentration was 10 µM, then the microplate was incubated for an additional 30 min at 37 °C. 

To determine the NO production, a 4-amino-5-methylamino-2′,7′-difluorofluorescein diacetate (DAF-FM) assay was performed according to the manufacturer’s instructions (Invitrogen, Carlsbad, CA, USA). DAF-FM fluorescent probe solution was added to each well to a final concentration 5 μM, then the microplate was further incubated for 40 min at 37 °C. In both cases, the fluorescence intensity was measured using a PHERAstar FS high-speed plate reader (BMG Labtech, Ortenberg, Germany) at λ_ex_ = 485 nm and λ_em_ = 518 nm. The data were processed using MARS Data Analysis v. 3.01R2 (BMG Labtech, Ortenberg, Germany). The results are presented as percentages of control with inductor data.

### 4.12. DPPH Radical Scavenging Activity

The 2,2-diphenyl-1-picrylhydrazyl (DPPH) radical scavenging activity of the peptides was tested as described in [88]. The peptides dissolved in MeOH were dispensed at a volume of 120 μL into the wells of a 96-well microplate. Then, 30 μL of the DPPH (Sigma-Aldrich, Steinheim, Germany) solution in MeOH (3.75 mM) was added to each well. The peptide concentration of the mixtures was 1 μM. The mixtures were shaken and incubated at room temperature for 30 min, and the absorbance of the resulting solutions was measured at 520 nm with a Multiscan FC microplate reader (Thermo Scientific, Waltham, MA, USA). The results are presented as percentages of the negative control (MeOH) data. Ascorbic acid at 10 μM was used as the positive control.

### 4.13. Expression of TRPV1 Channels in Xenopus Laevis Oocytes 

To express TRPV1 in *Xenopus laevis* oocytes, the linearized plasmids were transcribed using a T7 or SP6 mMESSAGE-mMACHINE transcription kit (Ambion, Austin, TX, USA). The harvesting of stage V–VI oocytes from anaesthetized female *X. laevis* frogs was carried out as previously described [89]. Oocytes were injected with 50 nL of cRNA at a concentration of 1 ng/nL using a micro-injector (Drummond Scientific, Broomall, PA, USA). The oocytes were incubated in a solution containing 96 mM NaCl, 2 mM KCl, 1.8 mM CaCl_2_, 2 mM MgCl_2_, and 5 mM HEPES pH 7.4, supplemented with 50 mg/L gentamicin sulfate.

### 4.14. Electrophysiological Assay

The physiological activity in oocytes heterologously expressing the TRPV1 channels was measured using the two-electrode voltage–clamp technique, using a Geneclamp 500 amplifier (Molecular Devices, Austin, TX, USA) controlled by the pClamp database system (Axon Instruments, Union City, CA, USA). The measurements were performed at room temperature (18–22 °C). Whole-cell currents were recorded 1–4 days after the mRNA injection. The electrode resistance was 0.7–1.5 MΩ. The signal was amplified and preliminarily filtered using an amplifier-embedded Bessel filter (cutoff frequency 500 Hz) after digitization of the signal at 2000 Hz. Recordings obtained before the activation of the examined currents were used to subtract the capacitive and leakage currents. The cells were kept at a holding potential of −90 mV. TRPV1 currents were measured in ND96 solution using a protocol of −90 mV for 400 s. The recording chamber was perfused at a rate of 2 mL/min with the ND96 solution. Capsaicin (2 µM) was used as an agonist and capsazepine (10 µM) was used as an antagonist of TRPV1. Capsaicin and capsazepine were purchased from Sigma-Aldrich (St. Louis, MO, USA). The use of the *X. laevis* animals was in accordance with the license number LA1210239 of the Laboratory of Toxicology and Pharmacology, University of Leuven (Belgium). The use of *X. laevis* animals was approved by the Ethical Committee for Animal Experiments of the University of Leuven (P186/2019). All animal care and experimental procedures agreed with the guidelines of the European Convention for the protection of vertebrate animals used for experimental and other scientific purposes (Strasbourg, 18.III.1986).

### 4.15. YO-PRO-1 Uptake Measurements

For the uptake measurements of the large cationic dye YO-PRO-1, the peptides at final concentrations of 0.01, 0.1, and 1.0 μM were added to the culture medium with Neuro-2a cells and further incubated for 1 h at 37 °C with 5% CO_2_. Then, the cells were washed once with Hanks’ balanced salt solution (HBSS) (140 mM NaCl, 5 mM KCl, 0.8 mM MgCl2, 2 mM CaCl2, 10 mM glucose, 10 mM HEPES, pH 7.4) and filled with 180 μL of the same buffer. YO-PRO-1 (Sigma-Aldrich, St. Louis, MO, USA) was loaded into the wells to a final concentration of 5 μM, the cells were incubated for 15 min at 37 °C, then ATP was added to a final concentration of 4 mM and the plates were incubated for an additional 10 min. Then, the cells were washed three times with the buffer solution and the fluorescence intensity levels were measured with a PHERAstar FS plate reader (BMG, Germany) at λ_ex_ = 480 nm and λ_em_ = 520 nm. A438079 (10 μM), a standard inhibitor of P2X7R, was used as positive control. The effectiveness of the peptides was evaluated relative to the control with ATP.

### 4.16. Ca^2+^ Influx Measurement

Neuro-2a cells were pre-incubated with studied peptides for 1 h at 37 °C with 5% CO_2_. Then, cells were washed twice with culture medium and loaded with 5 μM Fluo-8 dye (Sigma-Aldrich, St. Louis, MO, USA) and 1 μM Pluronic F-127 (Sigma-Aldrich, St. Louis, MO, USA) and incubated for 40 min at room temperature in HBSS saline at pH 7.4. Then, the cells were washed two times with the same solution but without the fluorescent dye and were incubated for 20–30 min at room temperature in the dark. In some experiments, Ca^2+^-free medium was used. Here, 140 mM NaCl, 5 mM KCl, 0.8 mM MgCl2, 10 mM glucose, 10 mM HEPES, 5 mM EGTA, pH 7.4. ATP (1 mM final concentration) were added using a robotic microinjector (20 μL/well), then 10 s later the baseline recording and additional readings were taken up to 50 s at 1 s intervals. The standard P2X7R inhibitor A438079 (Sigma-Aldrich, St. Louis, MO, USA) was used as the inhibitory control. Ionomycin (Sigma-Aldrich, St. Louis, MO, USA) was used to generate a generic calcium signal in cells. Fluo-8 was excited at 488 nm, and the emission at 520 nm was measured with a PHERAstar plate reader (BMG LABTECH, Ortenberg, Germany).

### 4.17. Surface Plasmon Resonance

SPR analyses were performed based on using a Biacore 3000 optical biosensor (GE Healthcare, USA) running under the program “Biacore 3000 Control Software v.1.0”. Recombinant human P2X7R (sequence positions of 47–334 a.a.; complete extracellular domain, 36.8 kDa) (Abbexa LTD, Cambridge, UK) was covalently immobilized on the carboxymethylated surface of the Biacore CM5 sensor chip (Cytiva, Chicago, IL, USA), then activated by the 1:1 mixture of 0.2 M 1-ethyl-3-(3-dimethylaminopropyl)carbodiimide hydrochloride (EDC) and 0.05 M N-hydroxysuccinimide (NHS) via the injection of receptor solution (15 μg/mL) in 10 mM sodium acetate (pH 5.0) for 10 min at a flow rate of 5 μL/min. The reference channel without immobilized P2X7R was used to correct the effects of the non-specific binding of peptides to the chip surface. The quantity of the immobilized hP2X7R equaled 12300 RU (resonance units, 1 RU corresponds to 1 pg receptor bound per mm^2^ of chip surface). HBS-N (10 mM HEPES, 150 mM NaCl, pH 7.4) (Cytiva, USA) was used as a running buffer for SPR assays. Peptide solutions in the HBS-N buffer over a concentration range of 5–100 μM were passed through biosensor channels (working and reference) at a flow rate of 10 µL/min for 3 min at 25 °C. Dissociation of the formed peptide–P2X7R complexes was registered at the same flow rate for no less than 6 min from the moment of sample injection. After each biosensor cycle, analytes were removed with two injections of regenerating solution (2 M NaCl, 1% CHAPS) at a flow rate of 30 μL/min for 30 s.

SPR sensorgrams were processed in BIAevaluation Software v. 4.1.1 (GE Healthcare) using “1:1 binding (Langmuir)” and “two-state (conformational change) binding” data processing models.

The final kinetic parameters were obtained from the model of two-state (conformational change) binding. The equation describing the used model was as follows:

*K_d_* = k_off1_/k_on1_ × (1 + k_on2_/k_off2_) − 1

where *K_d_* is the equilibrium dissociation constant, k_off1_ is the dissociation rate constant, k_on1_ is the association rate constant, k_on2_ is the forward rate constant for the CP ↔ CP * transition, k_off2_ is the backward rate constant for transition CP ↔ CP *, C is the compound, and P is the immobilized protein.

### 4.18. Statistics

All data were obtained from three independent replicates and calculated values were expressed as means ± standard error of the mean (SEM). Student’s *t*-test was performed using SigmaPlot 14.0 (Systat Software Inc., San Jose, CA, USA) to determine statistical significance.

## Figures and Tables

**Table 1:** The peptide secondary structural elements.

Sample	α-Helix			β-Structure			β-Turn	Unordered Structure
	I	II	III	I	II	III		
HCIQ2c1	7.1	13.9	21.0	17.4	6.9	24.3	19.0	35.7
HCIQ4c7	7.1	13.9	21.0	17.5	6.9	24.4	18.9	35.7
HCIQ1c9	10.6	16.2	26.8	14.2	5.7	19.9	19.3	34.0
HMIQ3c1	7.1	13.9	21.0	17.5	7.0	24.5	19.0	35.5
InhVJ	12.4	8.7	21.1	18.0	6.5	24.5	10.1	44.3
SHPI-1 *			20.0			21.8	18.2	40.0

I—Regular structure; II—irregular structure; III—summary value; * the data calculated using the CDSSR algorithm [54].

## Data Availability

The additional data supporting the manuscript are available from the corresponding author upon request.

## Supplementary Materials

The following supporting information can be downloaded at: https://www.mdpi.com/article/10.3390/ijms23095115/s1.

## Sample Availability

Samples of the recombinant peptides are available from the authors.
