# Supplementary figures and images for: Kunitz-Type Peptides from Sea Anemones Protect Neuronal Cells against Parkinson’s Disease Inductors via Inhibition of ROS Production and ATP-Induced P2X7 Receptor Activation
Source: Int J Mol Sci. 2022 May 4;23(9):5115. doi: 10.3390/ijms23095115 (PMC9103184; doi:10.3390/ijms23095115)

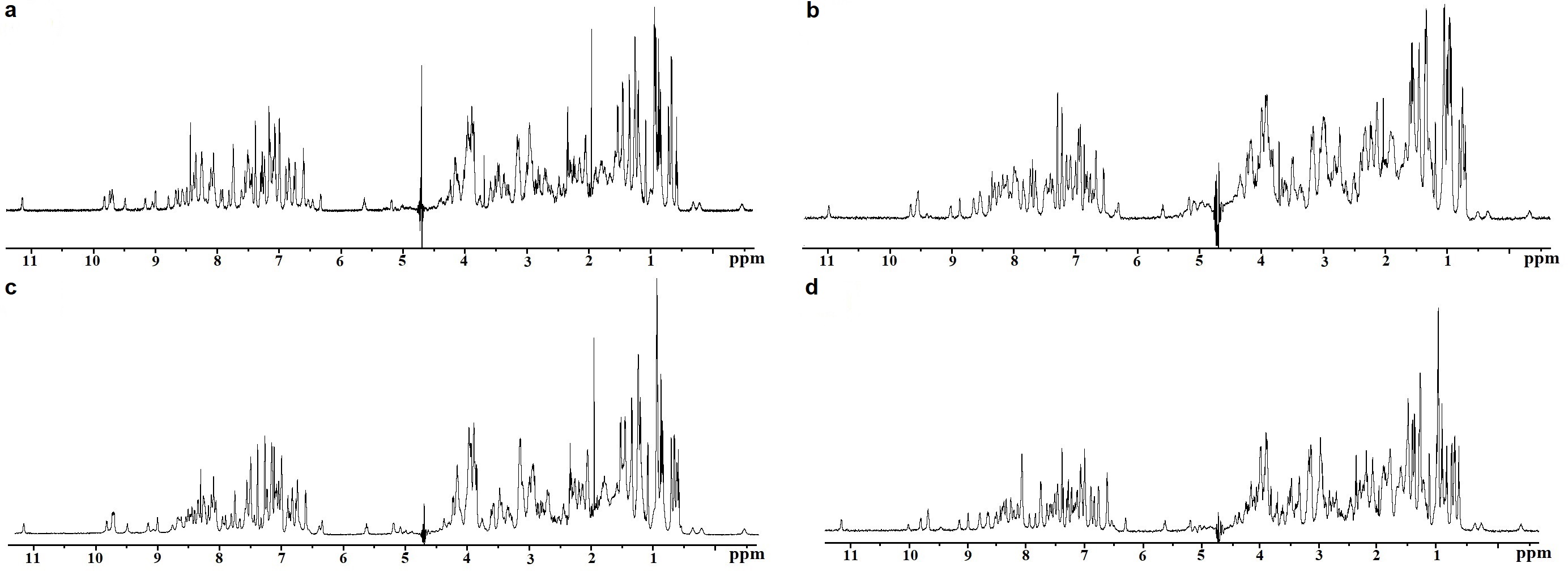

Supplement: Supplementary file 1 [file ijms-23-05115-s001.zip › Figure S1.jpg]

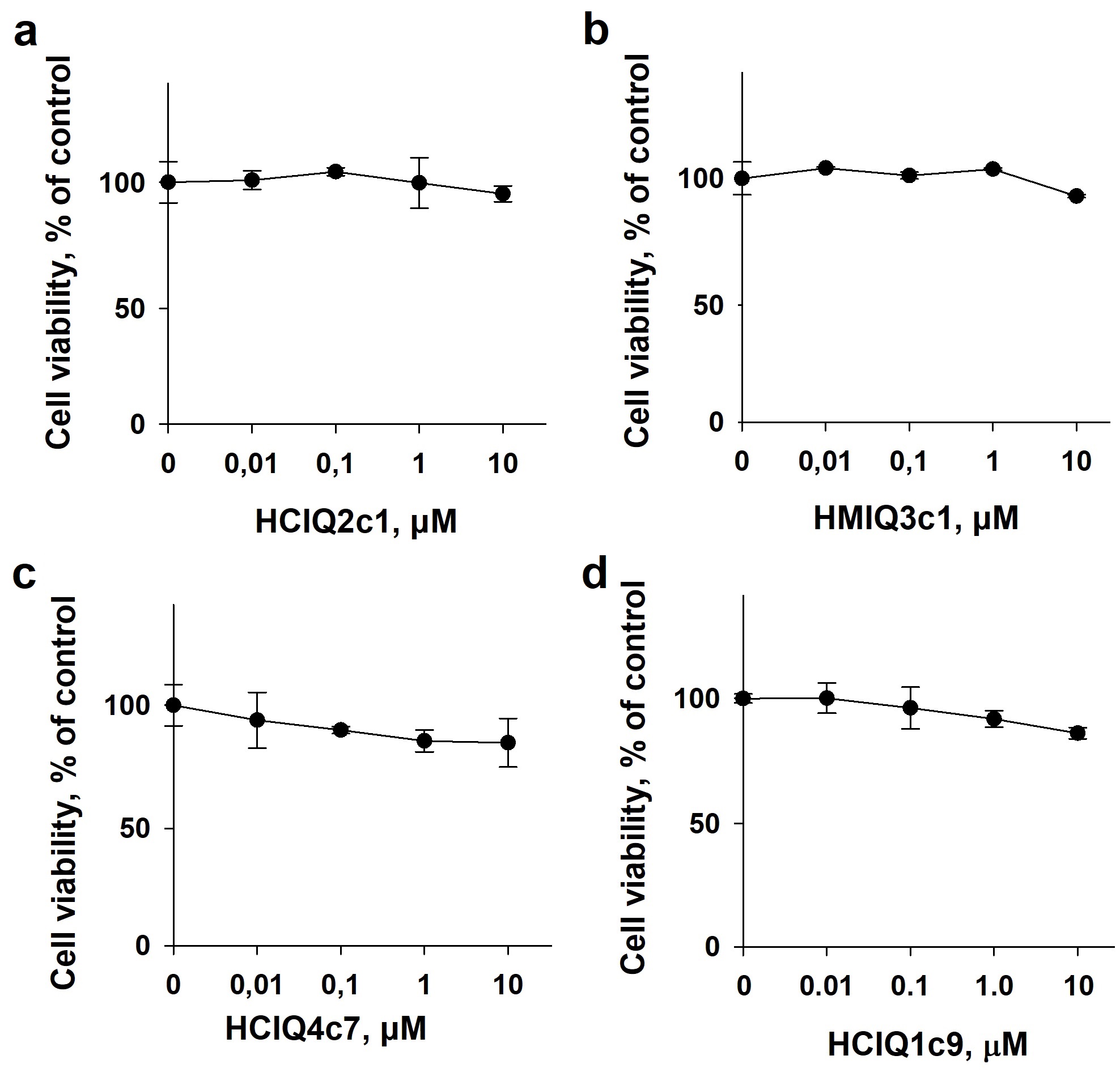

Supplement: Supplementary file 1 [file ijms-23-05115-s001.zip › Figure S2.jpg]
